# Estimating future heat-related and cold-related mortality under climate change, demographic and adaptation scenarios in 854 European cities

**DOI:** 10.1038/s41591-024-03452-2

**Published:** 2025-01-27

**Authors:** Pierre Masselot, Malcolm N. Mistry, Shilpa Rao, Veronika Huber, Ana Monteiro, Evangelia Samoli, Massimo Stafoggia, Francesca de’Donato, David Garcia-Leon, Juan-Carlos Ciscar, Luc Feyen, Alexandra Schneider, Klea Katsouyanni, Ana Maria Vicedo-Cabrera, Kristin Aunan, Antonio Gasparrini

**Affiliations:** 1 Environment & Health Modelling (EHM) Lab, Department of Public Health Environment & Society, London School of Hygiene & Tropical Medicine, London, UK; 2 Department of Economics, Ca’ Foscari University of Venice, Venice, Italy; 3 Division of Climate and Environmental Health, Norwegian Institute of Public Health, Oslo, Norway; 4 Doñana Biological Station, Spanish National Research Council, Seville, Spain; 5 Institute of Epidemiology, Helmholtz Zentrum München – German Research Center for Environmental Health, Neuherberg, Germany; 6 Department of Geography, University of Porto, Porto, Portugal; 7 Department of Hygiene, Epidemiology and Medical Statistics, National and Kapodistrian University of Athens, Athens, Greece; 8 Department of Epidemiology, Lazio Regional Health Service, ASL ROMA 1, Rome, Italy; 9 European Commission, Joint Research Centre, Seville, Spain; 10 European Commission, Joint Research Centre, Ispra, Italy; 11 Environmental Research Group, School of Public Health, Imperial College, London, UK; 12 Institute of Social and Preventive Medicine, University of Bern, Bern, Switzerland; 13 Oeschger Center for Climate Change Research, University of Bern, Bern, Switzerland; 14 CICERO Center for International Climate Research, Oslo, Norway

**Keywords:** Risk factors, Epidemiology

## Abstract

Previous health impact assessments of temperature-related mortality in Europe indicated that the mortality burden attributable to cold is much larger than for heat. Questions remain as to whether climate change can result in a net decrease in temperature-related mortality. In this study, we estimated how climate change could affect future heat-related and cold-related mortality in 854 European urban areas, under several climate, demographic and adaptation scenarios. We showed that, with no adaptation to heat, the increase in heat-related deaths consistently exceeds any decrease in cold-related deaths across all considered scenarios in Europe. Under the lowest mitigation and adaptation scenario (SSP3-7.0), we estimate a net death burden due to climate change increasing by 49.9% and cumulating 2,345,410 (95% confidence interval = 327,603 to 4,775,853) climate change-related deaths between 2015 and 2099. This net effect would remain positive even under high adaptation scenarios, whereby a risk attenuation of 50% is still insufficient to reverse the trend under SSP3-7.0. Regional differences suggest a slight net decrease of death rates in Northern European countries but high vulnerability of the Mediterranean region and Eastern Europe areas. Unless strong mitigation and adaptation measures are implemented, most European cities should experience an increase of their temperature-related mortality burden.

## Abstract (web summary)

Modeled analyses of 854 European cities show that net temperature-related mortality will increase because of an increase in heat-related mortality exceeding future reductions in cold-related mortality under current climate change projections and even in the presence of high adaptation scenarios.

## Main

Heat and cold are established health risk factors with a notable impact on mortality across Europe^1,2^. Estimates generally report that there are roughly ten cold-related deaths for each heat-related death^3–6^; some studies suggested that temperature-related mortality in Europe could overall decrease with climate change^7,8^. However, the balance between heat-related and cold-related mortality varies substantially across regions and over time with climate change. The latter has been associated with an important increase in heat-related deaths in the twenty-first century^2,9^. Increases in temperature are coupled with growth of urban areas and populations, which enhance exposure to high temperatures^10^. Given the current balance between heat-related and cold-related mortality burdens, the question of whether a decrease in cold exposure would offset the adverse increase in high heat exposure under climate change remains.

The balance between increased heat-related and decreased cold-related mortality, hereby referred to as the net effect of climate change, can be influenced by many factors. Previous studies provided inconsistent estimations of the net effect, depending on the location and considered scenarios^11–18^. Indeed, both extreme heat and cold change at different rates with climate change, resulting in narrowing or broadening of the temperature distribution depending on the region^19^. In addition, the exposure-response function (ERF) for temperature and mortality is complex, being usually U-shaped or J-shaped; it is often steeper on the heat side, although it varies widely between locations^4^. Previous evidence has been either too limited in scope or at a resolution too coarse to provide a representative impact estimate at the European level, while neglecting large portions of the continent, such as the Nordic and Baltic countries, and the Balkans^11–13,18^.

An additional complexity of projecting the net effect of climate change lies in the adaptive capacity of European populations. Several studies estimated a substantial attenuation of the heat risk on mortality over the last decades, generally linked to the increase in mean temperature or air conditioning penetration^20–22^; however, trends in cold-related mortality risks are less clear^22^. Despite some attempts at integrating heat adaptation into impact projections, through shifts in the minimum mortality temperature (MMT) or risk attenuation^13,15^, methodologies differ widely and with little empirical evidence to guide the modeling of adaptation. Adaptation to heat is additionally interlinked with underlying demographic and socioeconomic trends that necessitates integration within the shared socioeconomic pathways (SSP) framework^23,24^. Population aging results in increased vulnerability to both heat and cold^1,25,26^, while a general improvement in socioeconomic conditions and health systems under some SSP scenarios could, on the other hand, reduce the overall impacts that heat and cold have on mortality^27^. Given the complexities exposed above, projecting heat-related and cold-related mortality–and related net effect–under future conditions is inherently difficult because it depends on temperature projections from climate models and complex ERFs derived from epidemiological analysis, in addition to varying pathways in socioeconomic, demographic and vulnerability changes. An appropriate assessment of future temperature-related mortality must isolate the specific impact of climate change in a wide range of societal scenarios, while accurately propagating uncertainty from climate and epidemiological models.

In this study, we aimed to provide a comprehensive assessment of the net effect of climate change on temperature-related mortality across 854 cities spanning the whole European continent for the period 2015–2099 and for several levels of warming above preindustrial levels. We sought to provide insights on the expected evolution of the net effect in Europe, and under which conditions an increase of this net effect can be avoided. We evaluated a range of future demographic, mitigation and adaptation scenarios represented by a matrix of three SSP scenarios and four different heat adaptation scenarios.

## Results

### Study design

We considered three SSP scenarios based on European downscaling of the global scenarios and their effect on temperature-related adaptation^28^: (1) a more equitable Europe committed to sustainability and low-consumption lifestyles resulting in substantial action toward both mitigation and adaptation (SSP1-2.6); (2) a Europe maintaining current inequalities with increased privatization and slow progresses toward mitigation and adaptation (SSP2-4.5); and (3) a Europe with growing instability, regional conflicts and inequalities resulting in little to no effort toward mitigation and adaptation (SSP3-7.0). In each SSP scenario, we initially considered a baseline ‘no adaptation’ scenario in which the vulnerability to heat only depended on the local age distribution to provide a picture of the mortality burden of inaction toward adaptation to heat. We then evaluated a range of adaptation scenarios to heat by attenuating the heat-related mortality risk across ages by 10%, 50% and 90%. Attenuating the risk was done by shrinking the local age-specific ERF for temperatures above the MMT toward no association, according to the prespecified level.

This work builds on a published assessment of historical temperature-related mortality in 854 European urban areas with a population above 50,000, spanning a total of around 40% of 30 European countries^29^. We used the published city-specific ERFs derived for five age groups^1,30^, integrated them with projected temperature series and age-specific population and death rates for each SSP scenario, and performed comprehensive health impact projections^31^. In this assessment, we isolated the part specifically attributed to climate change by quantifying the burden as the difference in temperature-related deaths between two subscenarios: (1) ‘full’, in which both temperature and demographic projections are considered; and (2) ‘demographic change only’, in which only the demography changes while the temperature distribution from the period 2000–2014 is kept constant across the century. This allowed us to control for population aging and changes in mortality rates to isolate heat, cold and net effects of climate change directly attributable to the evolution of the temperature distribution and the population adaptation to heat. For each scenario described above, we accounted for climate uncertainty by considering bias-adjusted temperature outputs from 19 general circulation models (GCMs) extracted from the NASA Earth Exchange Global Daily Downscaled Projections database, based on the output from phase 6 of the Coupled Model Intercomparison Project (CMIP6)^32^. We additionally propagated the uncertainty from the epidemiological analysis by performing projections for 500 Monte Carlo simulations of the ERFs^30^. The methodology and the assumptions related to the several scenarios are fully detailed in the online methods and illustrated in the extended data.

### European-level results

**Fig. 1:**
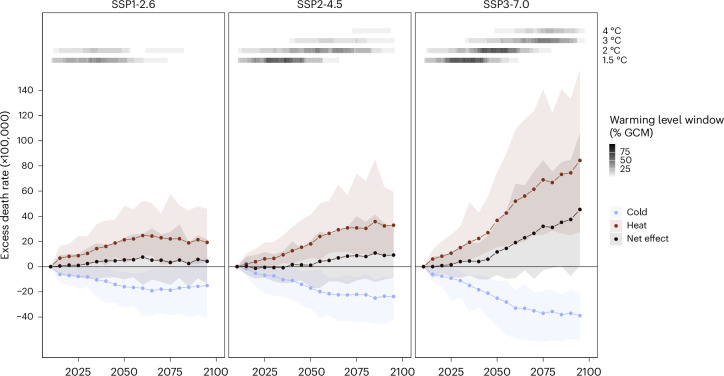
Projection of net changes in temperature-related excess death rates from 2015 to 2099 under no adaptation to heat for three SSP scenarios across 854 cities. The lines represent the average point estimate between the 19 GCMs considered. The transparent ribbons indicate the 95% empirical CIs from the 500 simulations in the 19 GCMs and the shades of gray at the top of each panel indicate the proportion GCM-specific warming level windows covering each year.

**Extended Data Fig. 1:**
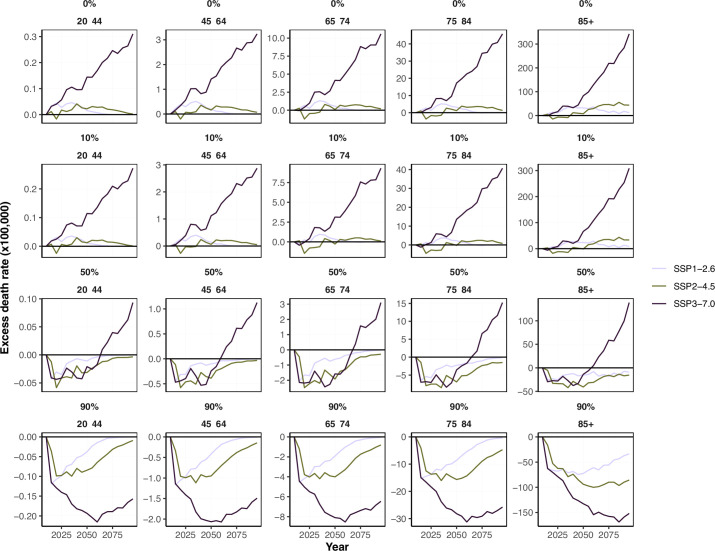
Projection of net temperature-related excess death rates from 2015 to 2099 under three SSP-RCP scenarios for each age group. Colored lines represent SSP scenarios, while panels separate adaptation scenarios (rows) and age groups (columns).

For the three considered SSP scenarios, the no adaptation scenario resulted in an increase in net temperature-related excess death rates, related to climate change only, across the whole 2015–2099 period (Fig. 1). In all cases, the increase in heat-related deaths outweighed the reduction in cold-related deaths, although the magnitude differed across SSP scenarios. For the SSP1-2.6 scenario, the net increase in temperature-related deaths peaked at 7.6 (95% confidence interval (CI) = −14.5 to 25.8) deaths per 100,000 person years in 2060, and decreased slightly afterward. In the SSP2-4.5 scenario, climate change-related death rates plateaued between eight and ten deaths per 100,000 person years from 2070 to the end of the century. In contrast, under the SSP3-7.0 scenario, the net effect substantially increased over the century to reach 45.4 (95% CI = 0.7 to 106.0) deaths per 100,000 person years (Table 1). This represents a 49.9% increase compared to the historical levels of 91 deaths per 100,000 person years^1,30^. Additionally, while temperature-related deaths almost disappeared for the youngest age groups under the SSP1-2.6 and SSP2-4.5 scenarios, rates consistently increased across all ages under SSP3-7.0 (Extended Data Fig. 1).

**Table 1:** Excess death rates (per 100,000 person years) in each country, region and at the European level for the periods 2050–2054 and 2095–2099 under the SSP3-7.0 and no adaptation to heat scenarios

	2050–2054			2095–2099		
	Cold	Heat	Net effect	Cold	Heat	Net effect
Finland	−19.0 (−44.5 to 5.4)	9.1 (−5.9 to 44.2)	−9.8 (−24.9 to 8.2)	−31.2 (−49.5 to −11.7)	28.2 (3.6 to 83.0)	−2.9 (−29.6 to 40.7)
Norway	−12.4 (−30.5 to −1.7)	6.2 (−0.4 to 22.8)	−6.2 (−18.0 to 6.7)	−23.1 (−39.9 to −9.0)	19.2 (2.2 to 58.6)	−3.9 (−24.1 to 27.9)
Estonia	−23.4 (−51.1 to 7.1)	9.7 (−9.6 to 50.8)	−13.7 (−33.6 to 9.9)	−42.8 (−70.0 to −15.2)	33.4 (3.9 to 103.2)	−9.4 (−45.0 to 47.5)
Sweden	−16.5 (−35.8 to −1.5)	9.2 (−0.3 to 40.5)	−7.3 (−21.2 to 13.0)	−26.9 (−42.5 to −10.9)	28.5 (3.2 to 79.5)	1.6 (−25.6 to 42.5)
Latvia	−30.2 (−65.3 to −1.7)	16.5 (−7.0 to 70.1)	−13.6 (−41.2 to 24.0)	−55.6 (−94.7 to −20.0)	46.8 (4.5 to 150.3)	−8.8 (−60.4 to 79.3)
Denmark	−17.7 (−44.3 to −1.8)	8.4 (−4.4 to 40.3)	−9.4 (−28.0 to 17.6)	−29.5 (−51.3 to −7.8)	25.0 (2.4 to 78.3)	−4.5 (−33.8 to 34.8)
Lithuania	−28.0 (−62.5 to −1.9)	15.5 (−6.2 to 66.9)	−12.5 (−39.9 to 25.1)	−48.8 (−86.4 to −17.8)	41.5 (4.2 to 136.8)	−7.3 (−54.5 to 75.2)
UK	−22.9 (−49.0 to −8.5)	10.9 (0.7 to 44.1)	−12.1 (−30.2 to 2.0)	−36.0 (−60.2 to −13.7)	31.2 (4.3 to 88.9)	−4.9 (−38.0 to 41.7)
Ireland	−33.5 (−67.7 to −13.3)	10.9 (0.4 to 48.9)	−22.6 (−51.9 to 6.1)	−56.5 (−95.2 to −21.8)	38.6 (2.4 to 143.7)	−18.0 (−65.7 to 72.5)
**Northern**	−**22.5 (**−**47.8 to** −**8.9)**	**10.7 (1.0 to 44.6)**	−**11.8 (**−**27.3 to 2.7)**	−**35.9 (**−**58.9 to** −**13.9)**	**31.0 (4.9 to 85.3)**	−**4.9 (**−**34.8 to 38.3)**
Netherlands	−16.1 (−36.7 to −1.1)	17.6 (2.2 to 62.2)	1.4 (−20.3 to 33.1)	−25.7 (−45.2 to −9.5)	44.4 (8.6 to 117.2)	18.7 (−21.3 to 81.7)
Germany	−16.4 (−35.8 to −2.9)	22.8 (1.8 to 64.2)	6.5 (−11.0 to 39.4)	−26.6 (−43.4 to −11.7)	63.6 (12.7 to 138.9)	36.9 (−8.1 to 103.8)
Belgium	−15.9 (−34.7 to −2.1)	17.7 (1.8 to 58.6)	1.8 (−17.2 to 30.3)	−27.0 (−44.5 to −11.1)	49.6 (10.6 to 117.2)	22.6 (−17.1 to 80.9)
Luxembourg	−11.3 (−26.2 to −2.3)	16.2 (0.9 to 51.3)	4.9 (−10.3 to 34.4)	−22.2 (−39.1 to −9.4)	54.8 (8.7 to 129.7)	32.6 (−10.3 to 105.1)
Austria	−15.3 (−33.2 to −2.8)	27.2 (3.8 to 73.6)	11.8 (−10.4 to 52.8)	−27.5 (−44.9 to −13.0)	91.0 (26.0 to 212.7)	63.5 (4.7 to 176.2)
France	−17.9 (−35.8 to −5.8)	27.2 (8.4 to 60.2)	9.3 (−11.4 to 40.8)	−28.4 (−44.2 to −15.5)	66.4 (15.2 to 134.8)	38.0 (−8.0 to 101.2)
Switzerland	−13.6 (−35.4 to −1.8)	25.0 (4.7 to 70.5)	11.4 (−8.1 to 51.3)	−24.0 (−40.8 to −11.1)	77.9 (18.6 to 169.2)	53.9 (−0.6 to 140.8)
**Western**	−**16.7 (**−**35.1 to** −**3.9)**	**23.9 (5.7 to 61.7)**	**7.1 (**−**9.8 to 35.3)**	−**27.2 (**−**42.6 to** −**13.9)**	**63.2 (18.4 to 132.7)**	**36.0 (**−**3.9 to 98.5)**
Poland	−28.3 (−60.1 to −5.5)	24.8 (−0.8 to 74.8)	−3.5 (−28.4 to 36.6)	−49.9 (−79.0 to −23.0)	80.7 (20.7 to 191.4)	30.8 (−27.9 to 130.9)
Czechia	−22.6 (−48.2 to −6.3)	20.5 (2.0 to 56.6)	−2.1 (−22.8 to 30.4)	−42.7 (−68.8 to −21.1)	74.1 (18.0 to 177.9)	31.5 (−22.5 to 126.9)
Slovakia	−23.4 (−49.6 to −6.6)	23.5 (2.8 to 63.8)	0.1 (−21.3 to 33.6)	−42.2 (−65.8 to −20.5)	82.1 (22.9 to 194.3)	39.9 (−13.0 to 143.0)
Hungary	−25.9 (−55.5 to −9.5)	24.8 (2.7 to 66.9)	−1.1 (−22.9 to 30.9)	−49.2 (−75.9 to −24.7)	94.4 (28.2 to 216.2)	45.2 (−13.1 to 156.7)
Romania	−33.0 (−60.7 to −11.0)	39.4 (10.3 to 100.2)	6.5 (−22.5 to 48.6)	−63.7 (−95.7 to −28.5)	144.7 (30.5 to 284.4)	81.0 (−5.3 to 210.2)
Bulgaria	−30.2 (−54.2 to −6.3)	41.8 (11.0 to 89.3)	11.6 (−17.7 to 48.6)	−51.3 (−78.4 to −22.7)	129.9 (25.6 to 244.4)	78.6 (−1.3 to 182.6)
**Eastern**	−**28.4 (**−**56.9 to** −**9.5)**	**28.9 (6.0 to 73.9)**	**0.4 (**−**22.4 to 29.8)**	−**51.7 (**−**79.2 to** −**25.4)**	**98.7 (30.5 to 207.4)**	**47.0 (**−**9.4 to 146.2)**
Slovenia	−21.2 (−47.6 to −5.9)	44.5 (12.5 to 96.1)	23.2 (−6.4 to 62.4)	−34.5 (−56.9 to −16.3)	117.3 (42.1 to 234.4)	82.8 (14.9 to 193.3)
Croatia	−29.1 (−59.9 to −11.5)	54.9 (18.4 to 107.2)	25.8 (−7.5 to 67.3)	−53.7 (−83.3 to −27.0)	154.4 (60.7 to 280.7)	100.7 (17.2 to 217.6)
Italy	−36.5 (−68.7 to −17.8)	91.2 (36.4 to 165.8)	54.7 (5.6 to 117.4)	−52.7 (−78.1 to −29.4)	191.3 (69.3 to 312.3)	138.6 (25.0 to 247.3)
Portugal	−59.1 (−93.8 to −18.2)	76.3 (19.0 to 161.5)	17.3 (−40.6 to 91.2)	−78.1 (−119.7 to −31.7)	135.0 (34.0 to 252.9)	56.9 (−22.2 to 166.7)
Spain	−33.8 (−56.3 to −12.2)	80.3 (24.6 to 143.8)	46.4 (−0.1 to 108.4)	−50.1 (−75.3 to −24.0)	175.6 (64.4 to 305.7)	125.5 (31.2 to 249.6)
Greece	−30.7 (−56.0 to −5.1)	64.2 (20.4 to 131.3)	33.6 (−7.3 to 93.3)	−55.8 (−83.5 to −29.7)	175.4 (57.2 to 274.1)	119.6 (23.7 to 214.7)
Malta	−53.2 (−77.0 to −23.3)	200.8 (50.6 to 316.5)	147.6 (26.0 to 265.7)	−73.3 (−103.7 to −36.9)	341.8 (103.1 to 489.1)	268.6 (63.7 to 408.6)
Cyprus	−31.2 (−48.9 to −5.9)	63.9 (20.0 to 122.8)	32.7 (−3.2 to 84.2)	−61.2 (−87.5 to −32.5)	155.5 (59.9 to 262.8)	94.3 (9.3 to 197.8)
**Southern**	−**36.3 (**−**60.5 to** −**15.7)**	**82.2 (31.5 to 141.5)**	**45.9 (3.5 to 99.6)**	−**53.8 (**−**78.1 to** −**27.8)**	**177.8 (65.5 to 284.7)**	**124.0 (27.9 to 220.3)**
**Total**	−**25.0 (**−**47.3 to** −**11.4)**	**36.7 (15.4 to 75.0)**	**11.7 (**−**6.6 to 35.5)**	−**38.9 (**−**58.0 to** −**21.1)**	**84.3 (27.5 to 156.0)**	**45.4 (0.7 to 106.0)**

The table includes cold-related and heat-related deaths and the net effect. The parentheses indicate the 95% empirical CIs. Values in bold indicate regional and European totals.

Considering the respective climate and demographic trends in each SSP scenario, the numbers above imply that by the end of the century, and with no adaptation, climate change-related annual excess deaths due to nonoptimal temperatures in European urban areas could reach 7,826 (95% CI = −24,627 to 40,142) under SSP1-2.6, 17,856 (95% CI = −16,885 to 60,303) under SSP2-4.5 and 80,010 (95% CI= 1,312 to 186,821) under SSP3-7.0. Cumulative deaths between 2015 and 2099 would lead to a total burden of 616,798 (95% CI = −790,865 to 2,411,446) deaths for SSP1-2.6, 636,034 (95% CI = −957,325 to 2,354,502) deaths for SSP2-4.5 and 2,345,410 (95% CI = 327,603 to 4,775,853) deaths for SSP3-7.0.

**Fig. 2:**
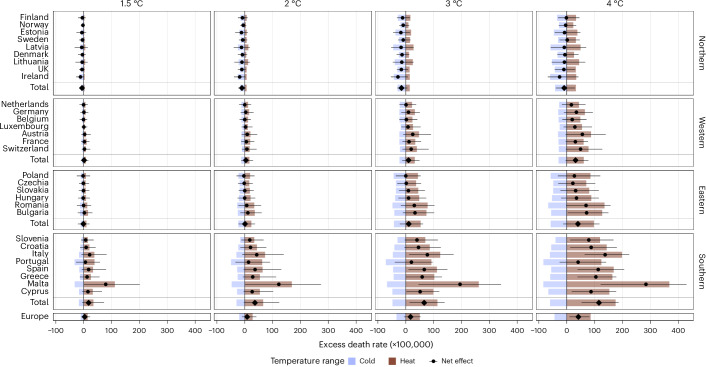
Country-level net changes in temperature-related excess death rates for each warming level under scenario SSP3-7.0 and no adaptation to heat. The bars and points represent the average point estimate between the 19 GCMs considered. The horizontal bars indicate the 95% empirical CIs of the net effect from the 500 simulations in the 19 GCMs. The diamond-shaped points indicate regional and European-level values. The temperature range is defined relative to the MMT.

Under SSP3-7.0, the net effect of climate change also increased exponentially with warming levels (Fig. 2), from 3.5 (95% CI = −8.6 to 22.7) deaths per 100,000 person years at 1.5 °C, to 17.2 (95% CI = −6.2 to 53.4) deaths per 100,000 person years at 3 °C and 41.7 (95% CI = 9.6 to 81.4) deaths per 100,000 person years at 4 °C. Considering the SSP3 demographic patterns, this amounts to 5,928 deaths per year (95% CI = −14,571 to 38,211) at 1.5 °C, 28,714 deaths per year (95% CI = −10,418 to 89,321) at 3 °C and 69,857 deaths per year (95% CI = 16,055 to 136,430) at 4 °C.

### Heterogeneity between countries

Results showed disparities between regions in the net effect of climate change, as illustrated by Fig. 2, which focuses on warming levels under SSP3-7.0 with no adaptation to heat. Southern Europe showed the largest net effects, reaching an increase of 124.0 (95% CI = 27.9 to 220.3) deaths per 100,000 person years by the end of the century (Table 1). Eastern and Western Europe presented net effects of climate change close to the European average, with respective increases of 47.0 (95% CI = −9.4 to 146.2) and 36.0 (95% CI = −3.9 to 98.5) deaths per 100,000 person years by the end of the century. With no adaptation to heat, only in Northern Europe the decrease in cold-related deaths slightly offsets the increase in heat-related deaths with a net effect of −11.8 (95% CI = −27.3 to 2.7) death per person years in 2050. This negative net effect was nonetheless more than halved at the end of century to −4.9 (95% CI = −34.8 to 38.3) deaths per 100,000 person years in 2095 (Table 1), and at 4 °C warming compared to 3 °C (Fig. 2).

There were also disparities within regions. The most affected country was the small island of Malta, showing a net effect of 268.6 (95% CI = 63.7 to 408.6) deaths per 100,000 person years in 2095. This was slightly more than twice the value for the Southern region, although with large uncertainty given the small population concerned. Ireland showed the lowest net effect of −18.0 (95% CI = −65.7 to 72.5) deaths per 100,000 person years by the end of the century and was the only country with almost no increase at the most extreme climate change scenarios. Romania (81.0, 95% CI = −5.3 to 210.2 deaths per 100,000 person years in 2095) and Bulgaria (78.6, 95% CI = −1.3 to 182.6 deaths per 100,000 person years in 2095) showed substantially higher net effects than other Eastern European countries.

### Spatial patterns

**Fig. 3:**
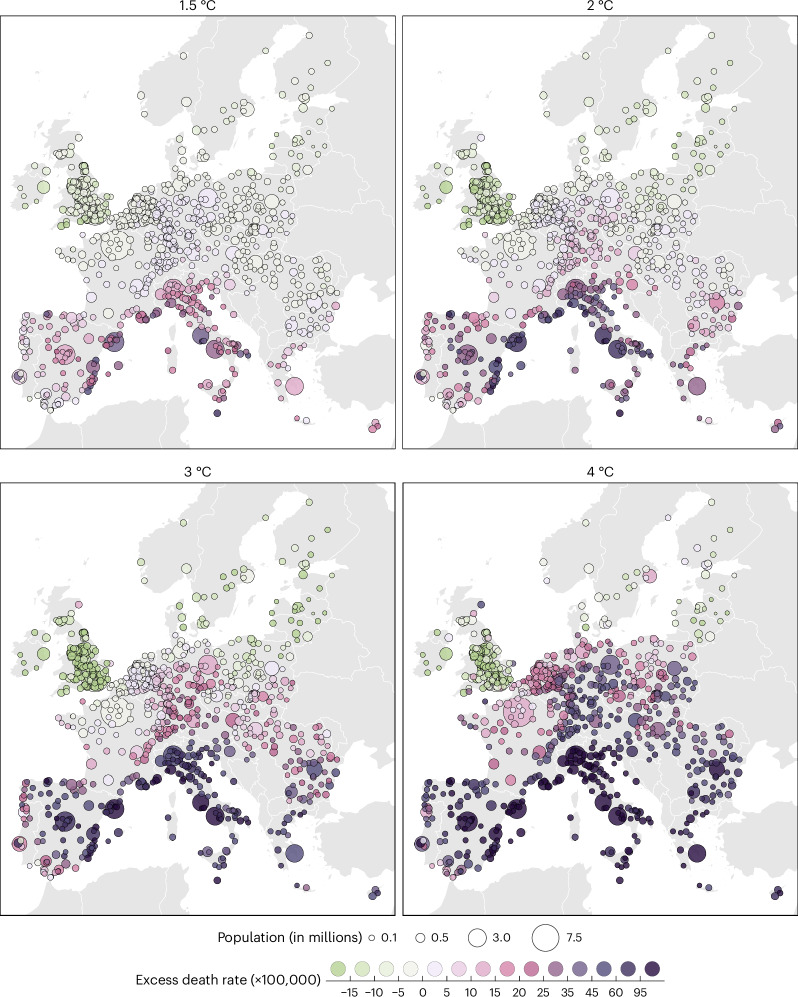
City-level net changes in temperature-related excess death rates for each warming level under scenario SSP3-7.0 and no adaption to heat. Panels from top-left to bottom-right represent warming levels from 1.5 °C to 4 °C. Green colors indicate a decrease of temperature-related excess deaths while purple colors indicate an increase.

Maps of excess death rates showed clear geographical patterns between and within countries (Fig. 3). There was a north–south gradient, especially in the Western part where differences were stronger between the UK and Spain, compared to differences between Finland and Bulgaria in the eastern part. There was also a strong Mediterranean effect, with the highest net effects in Eastern Spain, Southern France, Italy and Malta, which corresponds to a region in which the rate of climate warming is faster than in other areas. Finally, a central Europe hotspot can be seen, encompassing Switzerland and Austria (Fig. 2), as well as southern Germany and Poland.

Geographical disparities were stronger with global warming, especially between 1.5 °C and 3 °C. At a global mean temperature increase of 4 °C, the net effect substantially increased in Central Europe and reversed to become positive in some Northern cities such as Aberdeen, Stockholm, and Helsinki, and cities in the Balkan states and northern Poland.

### The role of heat adaptation

The results shown above provide a picture of the potential impacts of climate change if no action is taken toward adaptation to heat and cold. However, there is compelling evidence of variations in vulnerability to temperature across time, in particular to heat^21,22,33,34^. This section explores how the balance between increased heat-related and decreased cold-related mortality would change with various degrees of adaptation to heat.

**Fig. 4:**
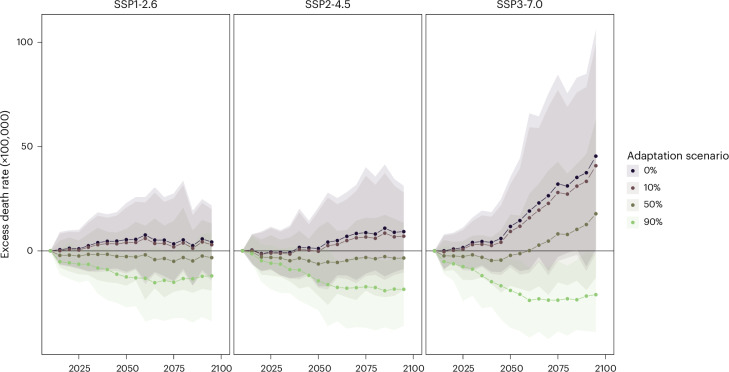
Projection of net changes in total temperature-related excess death rates from 2015 to 2099 under three SSP scenarios and four heat adaptation levels across 854 cities. The lines represent the average point estimate between the 19 GCMs considered. The transparent ribbons indicate the 95% empirical CIs from the 500 simulations in the 19 GCMs. The black lines (0% adaptation) correspond to the same black lines in Fig. 1.

Figure 4 shows that a 10% attenuation of the heat-related mortality risk would result in little decrease in the net effect of climate change across all SSP scenarios. A stronger attenuation of 50% would be enough to result in a net decrease in temperature-related mortality under SSP1-2.6 and SSP2-4.5, especially in the second half of the century, but not under SSP3-7.0. In the latter scenario and with an attenuation of 50%, the excess death rate would still increase by 17.8 (95% CI = −12.9 to 63.2) deaths per 100,000 person years (19.6% of the historical level) by the end of the century. This corresponds to a cumulative toll of 268,100 deaths (95% CI = −1,099,852 to 1,788,228). An almost complete attenuation of the heat-related mortality risk (90% decrease) would be needed to obtain a complete reversal of the net effect of climate change. This reversal is substantial under SSP3-7.0, in which the cold-related burden would greatly decrease.

**Extended Data Fig. 2:**
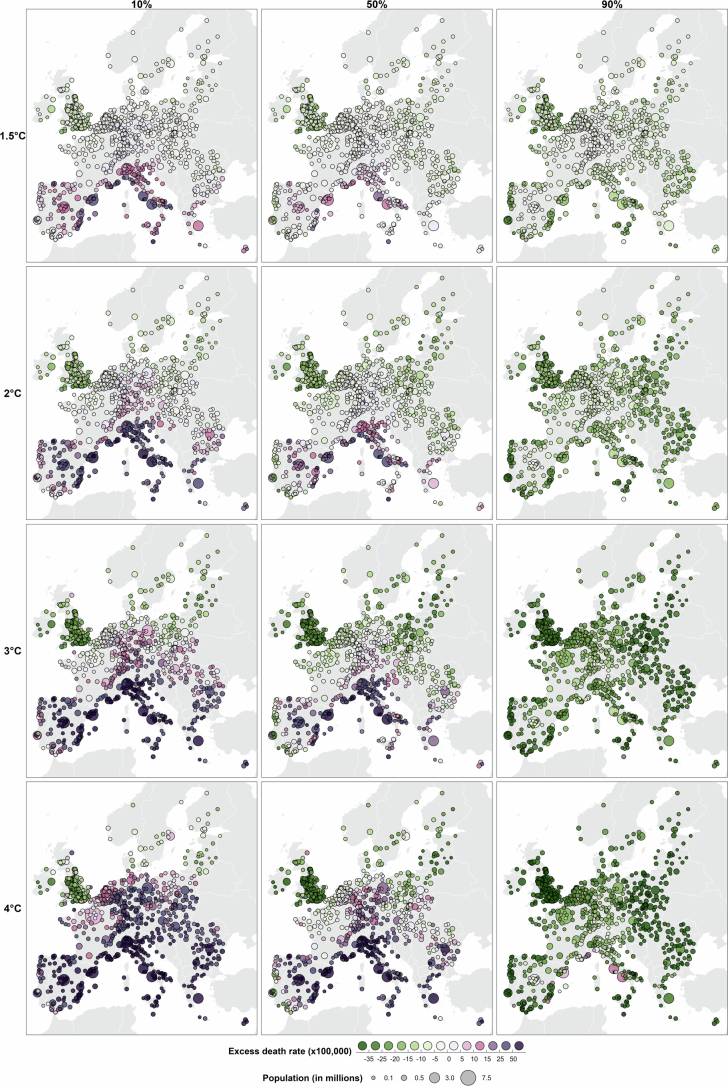
Estimated net effect of climate change at each warming level for each adaptation scenario under the SSP3-7.0 scenario. Panels separate warming levels (rows) and adapation scenarios (columns). Green colors indicate a decrease of net temperature-related deaths, and purple colors an increase.

There were important spatial disparities in the effects of adaptation (Extended Data Fig. 2). Specifically, even with a 50% attenuation of the heat-related mortality risk, most of the Mediterranean region would still experience a substantial increase in temperature-related mortality under SSP3-7.0. At high warming levels (3 °C and 4 °C), this persistence of a net effect above zero also spread to central Europe and some parts of the Balkans. Finally, it is worth noting that, even with an attenuation of 90%, a warming of 4 °C above preindustrial levels would still result in a net effect above zero for some cities in the Mediterranean region (among which Rome, Naples, Barcelona and Marseille).

## Discussion

In this study, we provided an extensive comparative assessment of changes in heat-related and cold-related mortality under several climate change, demographic and adaptation scenarios across European urban areas. We showed that, with no adaptation to heat, temperature-related mortality is expected to increase in response to climate change across scenarios and periods for most of Europe. For the selected 854 cities, this net effect could lead to a 50% increase in temperature-related death rates and a total cumulative death toll exceeding 2,000,000 toward the end of the century if the world were to follow the most extreme SSP3-7.0 scenario. These figures could be reduced by at least two-thirds under the more stringent SSP1-2.6 and SSP2-4.5 scenarios, outlining the health benefits of implementing strong policies to reduce carbon emissions.

Our results reveal that in the absence of ambitious mitigation, a substantial and rather implausible level of adaptation to heat is necessary to prevent increases in temperature-related mortality. Indeed, in the SSP3-7.0 scenario, a risk attenuation of 50% is not enough to compensate for the increased heat exposure due to climate change, in particular around the Mediterranean region, Central Europe and the Balkans. To put it in context, a 50% reduction in heat-related risk is higher than what has been observed in Germany^13^ and roughly in line with the decrease in risk that has been estimated in Spain over the last few decades (between 30% and 60%)^21,22,34^. Higher heat risk attenuation has only been estimated in countries like Sweden and Switzerland, which generally contribute much less to the heat-related mortality burden^22,35,36^. On the other hand, countries such as the UK, Greece and Czechia have seen little to no risk attenuation^21,37,38^. This shows that even ambitious objectives regarding adaptation can hardly replace feasible mitigation targets, especially in the context of SSP3-7.0.

Our results highlight spatial disparities with substantial future impacts in Mediterranean countries, and lower net increases of temperature-related deaths in Eastern and Western countries. Malta is the only country with estimated net increases in temperature-related deaths across all SSP and heat adaptation scenarios; the net death rate increase could reach up to almost 200% of the early twenty-first century levels. The increase could also exceed 80% in Spain. In addition to the Mediterranean region, we observed hotspots of net increases in temperature-related deaths in South-East European countries like Romania and Bulgaria, and in Central Europe, including Austria, southern Germany and southern Poland. These areas also correspond to countries in which the necessary heat risk attenuation to avoid an increase in temperature-related mortality is the highest. Overall, the spatial disparities were in line with several country-specific assessments^12,14,39^, although a one-to-one comparison is difficult given the differences in methodologies. Our results also suggest a slight net reduction in temperature-related deaths in northernmost countries, in line with previous assessments^11,17^. This slight reduction in temperature-related mortality nonetheless showed a reversal toward the end of the century and under more extreme warming scenarios, and was massively outweighed by the large increases in the rest of Europe.

The general net increase in temperature-related deaths across scenarios is related to the general shape of ERFs for temperature-related mortality^30^. Indeed, in most locations, the curve is steeper at high temperatures than at low temperatures, implying that for a given shift in the temperature distribution, the mortality risk increases more rapidly on the hot part than it decreases in the cold part. This also explains the necessity for strong attenuation of the heat risk in adapting to climate change. This effect is further exacerbated by the location of the local minimum mortality percentile as locations with smaller net increases tend to be locations with higher minimum mortality percentiles^1^. The reported net increase was not related to population increase and aging as our results report the difference between scenarios with and without climate change under the same population and baseline mortality trajectories. Therefore, we controlled for demographic effects by comparing two identical populations.

**Extended Data Fig. 3:**
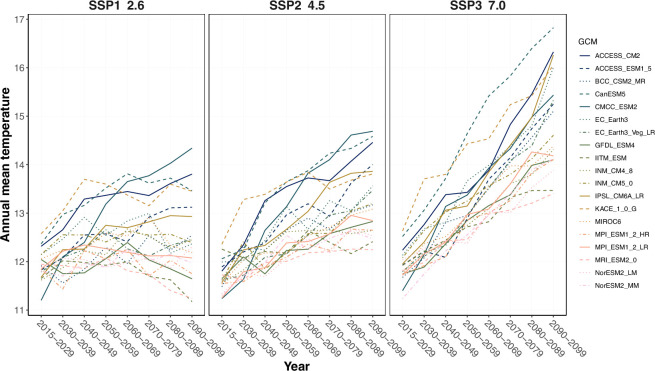
Average European five-year period temperature for each considered GCM. Panels represent the three considered SSP scenarios.

This study has several strengths. First, it provides a comprehensive assessment of the urban population in Europe, extending the analysis to several regions excluded in previous studies^11,17^, such as the Scandinavian, Balkan and Eastern European countries, for which evidence was almost nonexistent. Second, it made use of ERFs specific to each city and five age groups, whereas previous studies considered a single ERF for all ages^11,14,17^ or a single split at 75 years^12,13^. This allowed a better representation of the baseline death rate and vulnerability to heat and cold under different possible scenarios. Third, this study explored how the future net effect of climate would evolve under several scenarios of heat risk attenuation across age groups, thus providing a wider picture of climate change impacts. Finally, this study also improves the accuracy of the representation of the burden attributed to climate change and in the uncertainty of projections. This is done by isolating the specific impact of climate change on future mortality while still considering future demographic changes, unlike previous studies in which demographic change was either not accounted for^11,12,17^ or entangled with climate change in the results^13,15^. As climate projections are an inherently uncertain exercise, these projections integrated uncertainties both from the ERF estimation with the Monte Carlo simulations, and from the climate projections with many GCMs covering a wide range of possible futures (as illustrated in Extended Data Fig. 3). This uncertainty is also reflected in the results at warming levels, where previous studies chose a single year for each level^12,14^.

Despite the consideration of several risk attenuation scenarios, this work is limited in its treatment of adaptation. Indeed, scenarios were defined as general adaptation without geographical difference in the level of risk attenuation and without pointing to specific drivers of adaptation. The drivers of adaptation considered vary between studies, including temperature^15^, gross domestic product^13^, air conditioning^16^ or housing^40^; no consensual methodology on integrating these drivers in the adaptation has emerged. Additional local climate characteristics, such as humidity or specific hot night events, could impact temperature-related mortality and are sometimes integrated into projections^41,42^. However, epidemiological evidence is uncertain concerning the role of such factors and has suggested that nonlinear ERFs of mean temperature with adequate lags capture most of temperature-related mortality^43–46^. Therefore, future research should focus on understanding the interplay between local factors and vulnerability to temperature and on how to quantitatively integrate it into projections of temperature-related mortality.

The geographical scale of analysis at the city level considered in the present study also comes with some limitations. Indeed, temperatures and vulnerability to heat and cold can vary widely in a city^47,48^, which means that the most extreme temperatures and their potential impact may be smoothed out by our study design. Further projections accounting for within cities differences in exposure are needed to shed light on the most vulnerable populations. In addition, the definition of city, although extracted from Eurostat, which is a standardized database^49^, can differ according to the underlying administrative area in each country.

In conclusion, this study addresses open questions in climate change epidemiology, specifically about whether the increase in heat-related mortality will be offset by a reduction in cold-related deaths, and about the role of adaptation in future temperature-related health impacts^7,39,50^. Our study, based on a comprehensive assessment of 854 European cities, provides clear evidence that net mortality will increase even under the mildest climate change scenario. Our analyses indicate that the net health burden will increase substantially under more extreme warming scenarios and that this trend can only be reversed with implausibly strong levels of adaptation in urban populations. This demonstrates the potential health benefits linked with the implementation of stringent mitigation strategies to strongly reduce greenhouse gas emissions as well as adaptation strategies aimed at the most vulnerable countries and population groups.

## Methods

### Study area and period

The health impact projections were performed for 854 cities extracted from Eurostat’s Urban Audit project^51^, corresponding to all urban areas with more than 50,000 inhabitants in the EU-27, Norway, Switzerland and the UK. In total, these 854 cities account for around 40% of the population from the 30 countries considered. In the following analysis, we considered the period 2000–2099 separated in two: (1) the historical period from 2000 to 2014, which serves as a baseline and roughly corresponds to the period on which the ERFs were estimated^1^; and (2) the projection period 2015–2099 corresponding to the future scenario period from the phase 6 of the CMIP6 (ref. ^52^).

### Climate, demographic and adaptation scenarios

We considered three combined SSP scenarios: (1) SSP1-2.6, which corresponds to a sustainable world in which global warming barely exceeds 1.5 °C at its peak; (2) SSP2-4.5, in which the world progresses slowly toward sustainability and in which global warming probably remains below 3 °C; and (3) SSP3-7.0, which corresponds to a world with increased regional rivalries in which sustainability and environmental concerns are given low priority and which should result in a global warming probably close to or above 4 °C. In each SSP scenario, we additionally considered four adaptation scenarios associated with risk attenuation of heat: (1) the baseline scenario with no risk attenuation; (2) a slight adaptation of 10% after recent evidence in Germany^13^; (3) a strong adaptation of 50% in which the risk of heat is halved; and (4) an almost complete adaptation of 90%.

### Data sources

**Extended Data Fig. 4:**
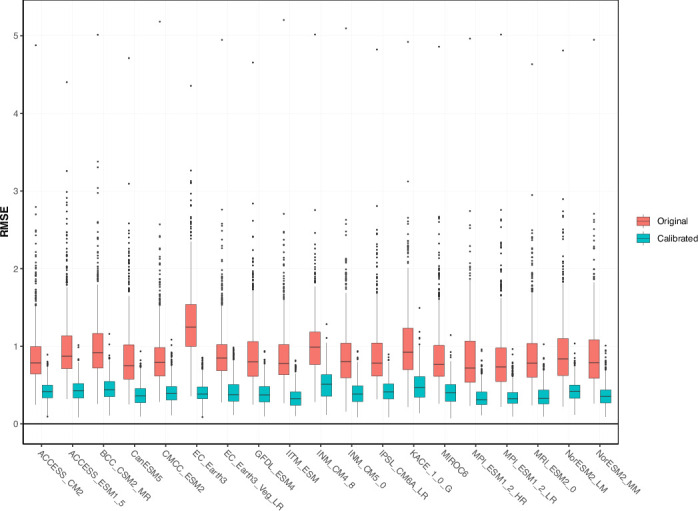
RMSE of the temperature distribution quantiles for the GCMs on the historical period compared to the observed ERA5-Land temperature quantiles. ‘Original’ refers to the GCM series as extracted from the NASA Earth Exchange Global Daily Downscaled Projections and ‘Calibrated’ refers to the calibrated GCM series. The box represents the interquartile range with the median as the middle segment and whiskers represent 1.5 times the interquartile range, of the RMSEs from the 854 cities and three SSP scenarios.

Extended Data Table 1 summarizes the different datasets used in this study. For each city, we retrieved ERFs for five age groups (20–44, 45–64, 65–74, 75–84 and 85+) from a previous health impact assessment^30^. For each SSP scenario and each city, we extracted projections of daily temperatures for 19 GCMs from the NASA Earth Exchange Global Daily Downscaled Projections database, which are downscaled and bias-corrected projections based on CMIP6 outputs^32^. To further align projected series to the observed series used in the historical assessment, we calibrated them on air temperature series extracted from the land component of the fifth generation of European ReAnalysis (ERA5-Land)^53^ using the Inter-Sectoral Impact Model Intercomparison Project bias adjustment and statistical downscaling (ISIMIP3BASD) method^54^, with the reference period being our historical period 2000–2014. Extended Data Fig. 4 illustrates the improved alignment with historical data after calibration, while Extended Data Fig. 3 shows the European average temperature across the century for each considered GCM. Years of exceedance of global warming levels at 1.5 °C, 2 °C, 3 °C and 4 °C were extracted from the Working Group I Atlas GitHub Repository for each considered GCM^55^. For each GCM and warming level, we considered a 20-year window around the provided year.

**Extended Data Fig. 5:**
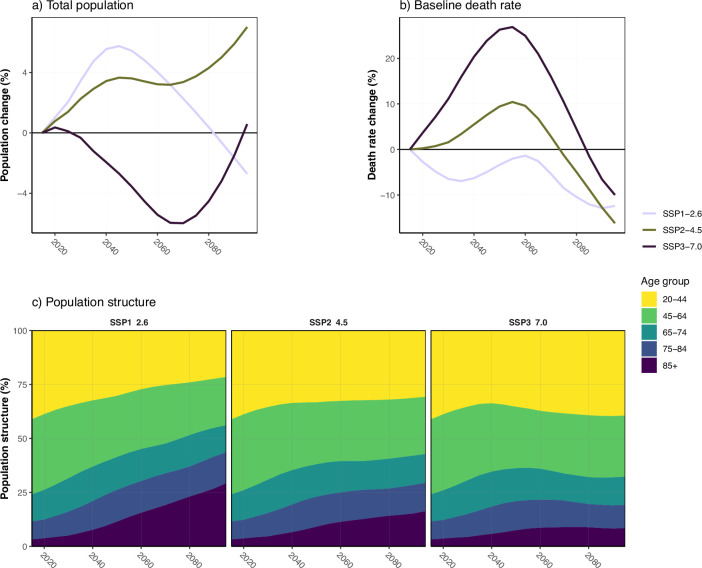
Projections of the European level demographic changes for each SSP. (**a**) population, (**b**) death rates and (**c**) population structure.

For each SSP, demographic projections at the country level were extracted from the Wittgenstein Centre Human Capital Data Explorer^56^. We extracted projections of population and survival ratios according to age group for each 5-year period. Projected deaths for 5-year periods were computed as one minus the survival ratio multiplied by the population at the start of the 5-year period. To obtain city-level projections, we computed the ratio between country-level values from the Wittgenstein Centre in the historical period 2010–2014 and city-level historical values extracted from Eurostat, and applied this correction factor to the projections. This method assumed that all cities in a country follow the same demographic trend. European-level demographic trends are shown in Extended Data Fig. 5.

### Statistical analysis

**Extended Data Fig. 6:**
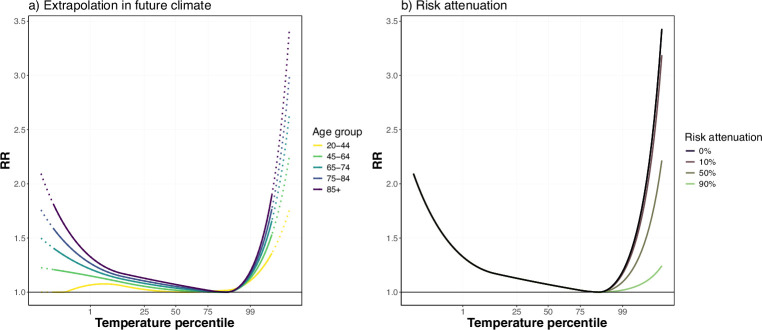
Average European exposure-response functions (ERF). (**a**) Age groups specific ERFs where dotted lines represent the extrapolated part on unobserved temperatures and the 20-44 ERF is truncated to 1 for extreme cold. (**b**) Illustration of heat-related mortality risk attenuation on the average European exposure-response function for age-group 85+.

Using projections of daily temperature, population and deaths, and the ERFs described above, we estimated the future number of deaths and death rates using a standard attribution methodology^1,17,57^. Briefly, for the whole period, the number of deaths attributed to temperature (attributable number (AN)) were computed for each city *i*, age group *a*, GCM *j* and day *t* using the standard formula:

1 $$\mathrm{A{N}}_{{ijat}}={d}_{{iat}}\frac{\left(\mathrm{R{R}}_{{ia}}\left({x}_{{ijt}}\right)-1\right)}{\mathrm{R{R}}_{{ia}}\left({x}_{{ijt}}\right)}$$

where $\mathrm{R{R}}_{{ia}}\left({x}_{{ijt}}\right)$ is the relative risk (RR) associated with the projected temperature ${x}_{{ijt}}$ for city *i*, GCM *j* and day *t* versus the MMT, which was extracted from the city *i* and age group *a*-specific ERF. For temperatures *x*_*ijt*_ outside the historical range, log-linear extrapolation was performed^58^. To avoid artifacts linked to extrapolation to outlier days far below the historically observed range, we constrained the RRs of temperature to never be below 1, as illustrated in Extended Data Fig. 6a. This is warranted for cities and age groups displaying slightly decreasing RRs at extreme cold values (see section D of the appendix). *d*_*iat*_ is the projected number of deaths for city *i*, age group *a* and day *t*, which were considered constant for each 5-year period.

For a specific adaptation scenario, the RR of temperatures above the local and age-specific MMT was attenuated by a factor *ϕ* as:^13^

2 $$\mathrm{R{R}}_{{ia}\phi }\left({x}_{{ijt}}\right)=1+\phi \left(\mathrm{R{R}}_{{ia}0}\left({x}_{{ijt}}\right)-1\right)$$

where *ϕ* represents the attenuation rate associated with each scenario (for example, 0.5 for 50% adaptation) and $\mathrm{R{R}}_{{ia}0}\left({x}_{{ijt}}\right)$ the RR in the baseline ‘no adaptation’ scenario. Risk attenuation at the European level is illustrated in Extended Data Fig. 6b.

**Extended Data Fig. 7:**
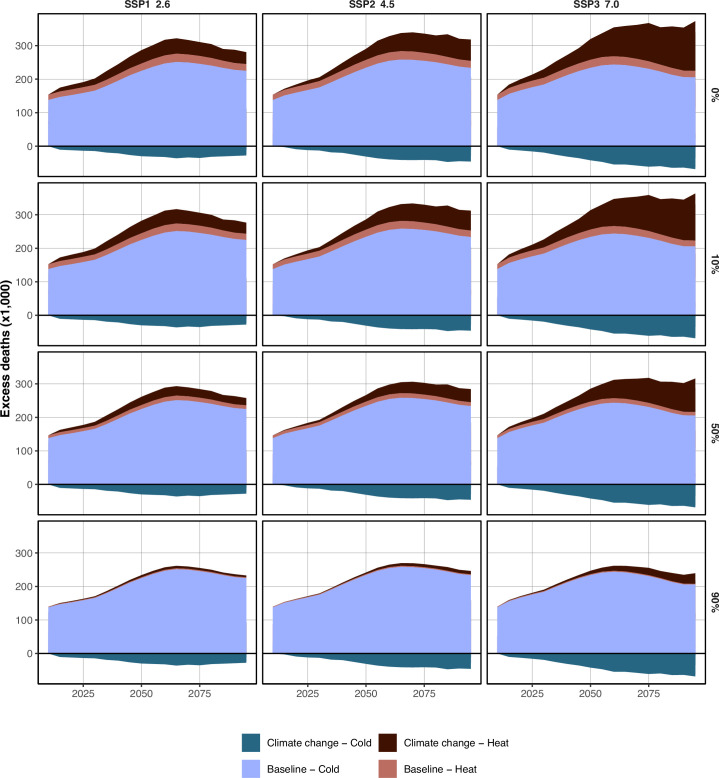
Decomposition of annual total excess deaths at the European level. The baseline components include the historical level and demographic change. Results reported in the main text correspond to the sum of the two dark colored areas.

To obtain climate change-related deaths, we computed ANs as described above under two subscenarios. In the ‘full’ subscenario, health impact projections were computed directly considering calibrated temperature and demographic projections as described by equation (1). In a second ‘demographic change-only’ subscenario, we performed health impact projections on a second set of temperature projections ${x}_{{ijt}}^{* }$, which were recalibrated such that the temperature distribution remained constant across the whole period. This recalibration was made by independently remapping the temperature quantiles of each decade to the historical temperature distribution using the ISIMIP3BASD bias correction method^54^ as if each decade was the historical period. This allowed to simulate a world without climate change but with the same demographic trends. We obtained the net effect of climate change $\mathrm{A{N}}_{{ijat}}^{{cc}}$ by differentiating these two subscenarios, thus allowing a comparison of two worlds with or without climate change but with the exact same populations (Extended Data Fig. 7).

To obtain the values reported in the article, we then summed climate change-attributed $\mathrm{A{N}}_{{ijat}}^{{cc}}$ across age group *a* and for 5-year periods and averaged the resulting values across GCM *j*. Excess death rates were obtained by dividing the aggregated $\mathrm{A{N}}_{{ip}}^{{cc}}$ by the projected population for the corresponding period. Warming level results were obtained by averaging daily values across the 21-year window surrounding the year of exceedance for each GCM. GCM-specific results for each warming level were then averaged. This allows transferring uncertainty of the demography at the timing of exceedance in the reported results for each warming level.

To further represent uncertainty, for each of the 19 GCMs, we performed health impact projections for 500 simulated ERFs extracted from the historical analysis^30^. CIs were then obtained as the 2.5% and 97.5% quantiles of the 9,500 computed death numbers and rates for each 5-year period and warming level.

### Reporting summary

Further information on research design is available in the Nature Portfolio Reporting Summary linked to this article.

## Online content

Any methods, additional references, Nature Portfolio reporting summaries, source data, extended data, supplementary information, acknowledgements, peer review information; details of author contributions and competing interests; and statements of data and code availability are available at 10.1038/s41591-024-03452-2.

## Supplementary information

Reporting Summary

## Figures and Tables

**Extended Data Table 1:**
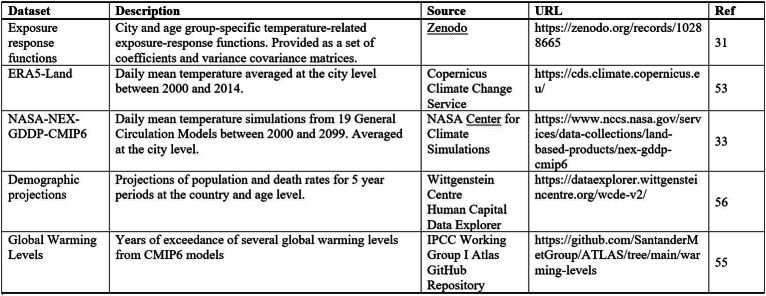
List of data used in the health impact projectionS

## Data Availability

The data used in this study and the results are publicly available in a *Zenodo* repository (10.5281/zenodo.14004322)^59^. The repository includes the R code used to extract all raw data for the analysis; links to individual data sources are indicated in Extended Data Table 1. Briefly, the city-level and age-group-level ERFs were extracted from a *Zenodo* repository (https://zenodo.org/records/10288665). Observed temperature series were obtained from the Copernicus Climate Change Service (https://cds.climate.copernicus.eu/), the projected temperature series from the NASA Center for Climate Simulations (https://www.nccs.nasa.gov/services/data-collections/land-based-products/nex-gddp-cmip6), demographic projections from the Wittgenstein Centre Human Capital Data Explorer (https://dataexplorer.wittgensteincentre.org/wcde-v2/) and the years of exceedance for global warming levels from the Intergovernmental Panel on Climate Change Working Group I Atlas GitHub Repository (https://github.com/SantanderMetGroup/ATLAS/tree/main/warming-levels).

## Code availability

The code used is fully reproducible and is available via GitHub and *Zenodo* (10.5281/zenodo.14004322)^59^.
