# Supplementary material for: Estimating future heat-related and cold-related mortality under climate change, demographic and adaptation scenarios in 854 European cities
Source: Nat Med. 2025 Jan 27;31(4):1294–302. doi: 10.1038/s41591-024-03452-2 (PMC12003192; doi:10.1038/s41591-024-03452-2)
Supplement: Supplementary file 1 — Reporting Summary [file 41591_2024_3452_MOESM1_ESM.pdf]

nature portfolio

Corresponding author(s): Pierre Masselot

Last updated by author(s): Oct 29, 2024

Reporting Summary

Nature Portfolio wishes to improve the reproducibility of the work that we publish. This form provides structure for consistency and transparency in reporting. For further information on Nature Portfolio policies, see our Editorial Policies and the Editorial Policy Checklist.

Statistics

For all statistical analyses, confirm that the following items are present in the figure legend, table legend, main text, or Methods section.

n/a	Confirmed	
☒	☐	The exact sample size (*n*) for each experimental group/condition, given as a discrete number and unit of measurement
☒	☐	A statement on whether measurements were taken from distinct samples or whether the same sample was measured repeatedly
☒	☐	The statistical test(s) used AND whether they are one- or two-sided<br>*Only common tests should be described solely by name; describe more complex techniques in the Methods section.*
☒	☐	A description of all covariates tested
☐	☒	A description of any assumptions or corrections, such as tests of normality and adjustment for multiple comparisons
☐	☒	A full description of the statistical parameters including central tendency (e.g. means) or other basic estimates (e.g. regression coefficient) AND variation (e.g. standard deviation) or associated estimates of uncertainty (e.g. confidence intervals)
☒	☐	For null hypothesis testing, the test statistic (e.g. *F*, *t*, *r*) with confidence intervals, effect sizes, degrees of freedom and *P* value noted<br>*Give P values as exact values whenever suitable.*
☒	☐	For Bayesian analysis, information on the choice of priors and Markov chain Monte Carlo settings
☐	☒	For hierarchical and complex designs, identification of the appropriate level for tests and full reporting of outcomes
☒	☐	Estimates of effect sizes (e.g. Cohen's *d*, Pearson's *r*), indicating how they were calculated

Our web collection on statistics for biologists contains articles on many of the points above.

Software and code

Policy information about availability of computer code

Data collection	Data have been collected and prepared using R 4.4.0. The code is fully available in a in a Zenodo repository (DOI: 10.5281/zenodo.14004322).
Data analysis	The analysis has been performed with R 4.4.0. The code is fully reproducible and publicly available through a Zenodo repository (DOI: 10.5281/zenodo.14004322).

For manuscripts utilizing custom algorithms or software that are central to the research but not yet described in published literature, software must be made available to editors and reviewers. We strongly encourage code deposition in a community repository (e.g. GitHub). See the Nature Portfolio guidelines for submitting code & software for further information.

Data

Policy information about availability of data

All manuscripts must include a data availability statement. This statement should provide the following information, where applicable:

- Accession codes, unique identifiers, or web links for publicly available datasets
- A description of any restrictions on data availability
- For clinical datasets or third party data, please ensure that the statement adheres to our policy

The data used in this study as well as the results are publicly available in a Zenodo repository (DOI: 10.5281/zenodo.14004322)60. The repository includes the R code extracting all raw data for the analysis, and links to individual data sources are indicated in Extended Data Table 1.
Briefly, the city and age-group level exposure-response function where extracted from a Zenodo repository (https://zenodo.org/records/10288665), observed

temperature series from the Copernicus Climate Change Service (https://cds.climate.copernicus.eu/), projected temperature series from the NASA Center for Climate Simulations (https://www.nccs.nasa.gov/services/data-collections/land-based-products/nex-gddp-cmip6), demographic projections from the Wittgenstein Cntre Human Capital Data Explorer (https://dataexplorer.wittgensteincentre.org/wcde-v2/) and years of exceedance for Global Warming Levels from the IPCC Working Group I Atlas GitHub Repository (https://github.com/SantanderMetGroup/ATLAS/tree/main/warming-levels).

Human research participants

Policy information about studies involving human research participants and Sex and Gender in Research.

Reporting on sex and gender	Information not available.
Population characteristics	No human data have been used in this study.
Recruitment	n/a
Ethics oversight	n/a

Note that full information on the approval of the study protocol must also be provided in the manuscript.

Field-specific reporting

Please select the one below that is the best fit for your research. If you are not sure, read the appropriate sections before making your selection.

☒ Life sciences ☐ Behavioural & social sciences ☐ Ecological, evolutionary & environmental sciences

For a reference copy of the document with all sections, see nature.com/documents/nr-reporting-summary-flat.pdf

Life sciences study design

All studies must disclose on these points even when the disclosure is negative.

Sample size	The 854 cities have been selected based on the Eurostat Urban Audit database, and correspond to all cities with more than 50,000 inhabitants.
Data exclusions	Overseas cities from Spain, Portugal, France and Iceland have been excluded due to different climatic conditions.
Replication	9,500 as a combination of 19 general circulation models (GCM) and 500 simulations from the exposure-response functions distribution.
Randomization	Observational study on aggregated datasets.
Blinding	Observational study on aggregated datasets.

Reporting for specific materials, systems and methods

We require information from authors about some types of materials, experimental systems and methods used in many studies. Here, indicate whether each material, system or method listed is relevant to your study. If you are not sure if a list item applies to your research, read the appropriate section before selecting a response.

Materials & experimental systems

n/a	Involved in the study
☒	☐ Antibodies
☒	☐ Eukaryotic cell lines
☒	☐ Palaeontology and archaeology
☒	☐ Animals and other organisms
☒	☐ Clinical data
☒	☐ Dual use research of concern

Methods

n/a	Involved in the study
☒	☐ ChIP-seq
☒	☐ Flow cytometry
☒	☐ MRI-based neuroimaging
